# Viral MicroRNA Effects on Pathogenesis of Polyomavirus SV40 Infections in Syrian Golden Hamsters

**DOI:** 10.1371/journal.ppat.1003912

**Published:** 2014-02-06

**Authors:** Shaojie Zhang, Vojtech Sroller, Preeti Zanwar, Chun Jung Chen, Steven J. Halvorson, Nadim J. Ajami, Corey W. Hecksel, Jody L. Swain, Connie Wong, Christopher S. Sullivan, Janet S. Butel

**Affiliations:** 1 Department of Molecular Virology and Microbiology, Baylor College of Medicine, Houston, Texas, United States of America; 2 Department of Molecular Biosciences, The University of Texas at Austin, Austin, Texas, United States of America; 3 Center for Comparative Medicine, Baylor College of Medicine, Houston, Texas, United States of America; University of Michigan, United States of America

## Abstract

Effects of polyomavirus SV40 microRNA on pathogenesis of viral infections *in vivo* are not known. Syrian golden hamsters are the small animal model for studies of SV40. We report here effects of SV40 microRNA and influence of the structure of the regulatory region on dynamics of SV40 DNA levels *in vivo*. Outbred young adult hamsters were inoculated by the intracardiac route with 1×10^7^ plaque-forming units of four different variants of SV40. Infected animals were sacrificed from 3 to 270 days postinfection and viral DNA loads in different tissues determined by quantitative real-time polymerase chain reaction assays. All SV40 strains displayed frequent establishment of persistent infections and slow viral clearance. SV40 had a broad tissue tropism, with infected tissues including liver, kidney, spleen, lung, and brain. Liver and kidney contained higher viral DNA loads than other tissues; kidneys were the preferred site for long-term persistent infection although detectable virus was also retained in livers. Expression of SV40 microRNA was demonstrated in wild-type SV40-infected tissues. MicroRNA-negative mutant viruses consistently produced higher viral DNA loads than wild-type SV40 in both liver and kidney. Viruses with complex regulatory regions displayed modestly higher viral DNA loads in the kidney than those with simple regulatory regions. Early viral transcripts were detected at higher levels than late transcripts in liver and kidney. Infectious virus was detected infrequently. There was limited evidence of increased clearance of microRNA-deficient viruses. Wild-type and microRNA-negative mutants of SV40 showed similar rates of transformation of mouse cells *in vitro* and tumor induction in weanling hamsters *in vivo*. This report identified broad tissue tropism for SV40 *in vivo* in hamsters and provides the first evidence of expression and function of SV40 microRNA *in vivo*. Viral microRNA dampened viral DNA levels in tissues infected by SV40 strains with simple or complex regulatory regions.

## Author Summary

The recent discovery of virally encoded microRNAs (miRNAs) raises the possibility of additional regulatory processes being involved in viral replication, immune recognition, and host cell survival. In this study, we sought to characterize the effect of SV40-encoded miRNAs and the structure of the viral regulatory region on infections in outbred Syrian golden hamsters. Results revealed that SV40 has a wide tissue tropism, including liver, kidney, spleen, lung, and brain, with kidney the preferred site for long-term persistent infection. Significant increases in tissue-associated viral DNA loads were observed with miRNA-negative mutant strains, whereas the presence of SV40 miRNAs had no effect on tumor induction and little effect on viral clearance. Our results provide the first evidence for SV40 miRNA expression and function in an *in vivo* animal model and highlight the complexity of regulation of SV40 viral replication and persistent infections.

## Introduction

Definition of steps in the pathogenesis of a viral infection and of the response of the host to the viral assault is important to understanding the genesis of associated diseases and to guiding the development of viral diagnostics, therapeutics, and preventative measures. Viral microRNAs (miRNAs) are predicted to be involved in the infection process.

Polyomaviruses are small, nonenveloped, DNA-containing viruses that establish persistent infections in susceptible hosts and induce tumors under certain conditions. The Polyomaviridae family contains several viral species able to infect humans, some of which have been linked with disease, especially in immunocompromised hosts. Little is known of the patterns and dynamics of acute and chronic infections by polyomaviruses *in vivo*, except that kidneys appear to be a common site of long-term persistence [1].

Simian virus 40 (SV40) is the type species of the virus family. Since its discovery in 1960, it has served as a model cancer virus and has revealed many insights into fundamental cell processes and basic mechanisms of cell transformation [2]–[8]. SV40, whose natural host is the rhesus macaque monkey, is able to infect humans. It is currently the only polyomavirus that can infect humans that has a small animal model amenable to mechanistic studies of early steps in viral pathogenesis. Syrian golden hamsters *(Mesocricetus auratus)* have been widely utilized for studies of SV40 infections and oncogenesis [9]–[17]. It has been shown that SV40 isolates differ in oncogenic potential *in vivo*, with those having a regulatory region (RR) with a single enhancer region being more oncogenic in weanling hamsters than those with a complex, rearranged RR. These RR differences, however, had no effect on transforming frequencies *in vitro*, suggesting that differences in tumor induction reflected viral interactions with the host [16]. Conversely, vertical transmission of virus from pregnant dams to their offspring was more frequent when infections involved an SV40 strain with a complex RR [15]. In addition, viruses with a complex RR induce more frequent antibody responses to the SV40 large T-antigen (T-ag) in tumor-free animals than those with a simple RR following intraperitoneal inoculation [10], [16]. We interpret these observations as indications that viruses with complex RR replicated better *in vivo*, increasing the likelihood of vertical transmission and of elevated synthesis of T-ag, the latter leading to more detectable T-antibody responses. Despite these findings, the effect of viral genetic variation among virus strains on the natural history of acute and persistent infections is not known.

miRNAs are small, noncoding RNAs that are key regulators of gene expression, able to control large networks of genes by binding and repressing mRNAs; miRNAs can regulate cell cycle genes and host response genes, modulate downstream gene expression, and play a role in tumorigenesis [18], [19]. SV40, the first polyomavirus found to encode viral miRNAs, expresses a precursor miRNA giving rise to two derivative effectors during infection in cultured cells. Both SV40 miRNA effectors (5p and 3p) reduce expression of the viral early proteins [20]. The presence or absence of viral miRNA appeared to have no effect on replication of the virus in tissue culture, although there were higher levels of T-ag in mutant-infected cells, and it was proposed that the miRNAs might function *in vivo* to help infected cells avoid killing by the host immune response. To date, no animal studies addressing SV40 miRNA function *in vivo* have been reported.

This project was undertaken to characterize patterns of acute and chronic infections by polyomavirus SV40 in a susceptible host. Specific objectives were (i) to identify tissues that can be infected by SV40 and those that are sites of long-term persistence, (ii) to measure quantitative changes in viral DNA levels over time in different tissues, (iii) to determine if SV40 genetic differences, including the presence of viral miRNA and the structure of the viral RR, affect the patterns of infections *in vivo*, and (iv) to evaluate the influence of viral miRNA on tumor induction.

## Results

### Frequent establishment of persistent infections by SV40

Young adult, outbred Syrian golden hamsters were inoculated by the intracardiac route with 1×10^7^ plaque-forming units (PFU) of two wild-type (WT) strains of SV40, one having a complex (2E) RR (776-WT) (GenBank accession No. J02400) and one a simple archetypal (1E) RR (SVCPC-WT) (GenBank accession No. AF156108), and with mutants unable to produce SV40 miRNAs derived from each WT strain (776-SM1, SVCPC-SM2).

The intracardiac route of inoculation was used to distribute the virus throughout the body in order to identify susceptible tissues. Animals were sacrificed at times ranging from 3 to 270 days postinoculation (p.i.), organs were harvested, and the presence of SV40 DNA was determined by real-time quantitative polymerase chain reaction (RQ-PCR) assays as detailed in Materials and Methods. The total numbers of tissues analyzed and the percentages that were positive for detectable levels of viral DNA are summarized (Table 1). All four viruses were efficient at establishing persistent infections. Animals were frequently virus-positive in liver, kidney, and spleen through day 45 and in kidney through day 270. In fact, all the liver and kidney samples collected through day 45 from infected animals were virus positive with only two exceptions. Spleen, lung and brain less commonly contained detectable viral DNA and virus disappeared more quickly. Muscle and cheek pouch specimens from 15 animals inoculated with the 776 viruses were tested at days 3, 7, and 45 p.i. and all were negative for viral DNA (data not shown).

**Table 1:** Frequent persistence of SV40 in hamster tissues following intracardiac inoculation.

Virus	Encode SV40 microRNA	Days p.i.	No. animals with SV40-positive tissues/No. tested (%)^a^				
			Liver	Kidney	Spleen	Lung	Brain
776-WT	+	3	4/4 (100)	4/4 (100)	4/4 (100)	4/4 (100)	2/4 (50)
		7	3/3 (100)	3/3 (100)	2/2 (100)	3/3 (100)	0/3 (0)
		14	4/4 (100)	4/4 (100)	2/4 (50)	0/3 (0)	0/4 (0)
		28	3/4 (75)	4/4 (100)	0/4 (0)	0/4 (0)	0/4 (0)
		45	3/3 (100)	3/3 (100)	ND	0/3 (0)	0/3 (0)
		270	3/7 (43)	6/7 (86)	ND	ND	ND
776-SM1	0	3	4/4 (100)	4/4 (100)	3/4 (75)	3/4 (75)	1/4 (25)
		7	4/4 (100)	4/4 (100)	4/4 (100)	1/4 (25)	1/4 (25)
		14	4/4 (100)	4/4 (100)	3/4 (100)	0/4 (0)	0/4 (0)
		28	4/4 (100)	4/4 (100)	0/3 (0)	ND	0/3 (0)
		45	3/3 (100)	4/4 (100)	2/4 (50)	1/4 (25)	0/3 (0)
		270	0/9 (0)	5/8 (62)	ND	ND	ND
SVCPC-WT	+	3	4/4 (100)	4/4 (100)	4/4 (100)	4/4 (100)	3/3 (100)
		7	4/4 (100)	4/4 (100)	4/4 (100)	1/4 (25)	0/3 (0)
		14	4/4 (100)	4/4 (100)	4/4 (100)	1/4 (25)	1/3 (33)
		28	4/4 (100)	4/4 (100)	4/4 (100)	0/4 (0)	1/3 (33)
		45	4/4 (100)	4/4 (100)	3/4 (75)	1/4 (25)	ND
		270	1/6 (17)	5/6 (83)	ND	ND	ND
SVCPC-SM2	0	3	4/4 (100)	4/4 (100)	4/4 (100)	4/4 (100)	3/4 (75)
		7	4/4 (100)	4/4 (100)	3/3 (100)	4/4 (100)	1/3 (33)
		14	3/3 (100)	2/2 (100)	4/4 (100)	0/3 (0)	1/3 (33)
		28	4/4 (100)	3/4 (75)	3/4 (75)	1/4 (25)	0/1 (0)
		45	4/4 (100)	4/4 (100)	2/4 (50)	3/4 (75)	0/2 (0)
		270	1/6 (17)	8/8 (100)	ND	ND	ND

ND, not done; p.i., postinoculation; SV40, simian virus 40.

a A tissue was considered positive if viral DNA copies were detected by real-time quantitative polymerase chain reaction (described in Materials and Methods).

Polyomaviruses are known to establish persistent infections and these results illustrate the highly efficient nature of the process by SV40 (Table 1). At this level of analysis in which tissue samples were either positive or negative for viral DNA, no obvious effect of viral miRNA or the viral RR on the dynamics of viral presence in different tissues could be discerned.

### Distribution of viral loads among infected animals and tissues

**Figure 1:**
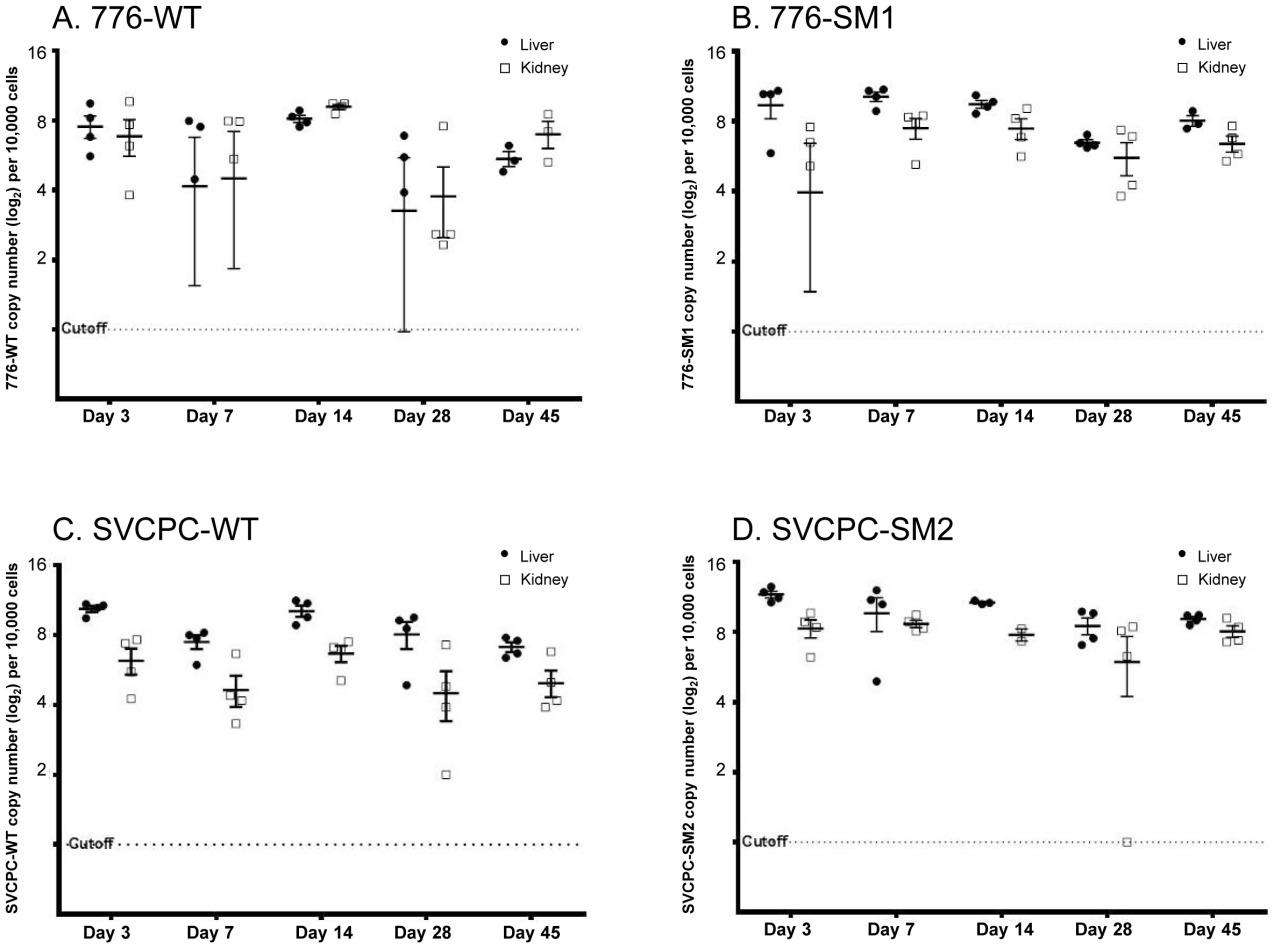
Simian virus 40 viral loads in hamster liver and kidney over time. Observed (nonadjusted) viral DNA copies per 10^4^ cells (log_2_) are presented for both liver and kidney specimens from individual animals. The geometric mean for each set of animals is marked by a horizontal line. Values are shown for days 3 to 45 postinoculation. (A) 776-WT. (B) 776-SM1. (C) SVCPC-WT. (D) SVCPC-SM2.

Viral DNA levels in hamster tissues were quantitated using an SV40-specific RQ-PCR assay. The single-copy hamster vimentin gene (GenBank accession No. AH001833.1) was also measured by RQ-PCR to determine the number of cell equivalents of DNA in tissue extracts; viral loads were then expressed as SV40 DNA copies per 10^4^ cells. Observed viral loads (log_2_) in liver and kidney samples from individual animals are shown for each of the four viruses (Figure 1). Geometric mean values for each set of experimental animals are represented by horizontal lines. Decreases in viral loads tended to occur between days 14 and 28 with all four viruses, although viral DNA remained readily detectable. There was often a range in viral loads among the animals within a virus group harvested at each time point, presumably reflecting at least in part individual variation typical of outbred animals.

The relative amounts of virus found in liver, kidney, spleen, lung, and brain early during infection (days 7 and 14) are shown (Table 2). Data for the four viruses are presented as averages of observed values (SV40 DNA copies/10^4^ cells). During the early stages of infection SV40 viral loads were higher in liver and kidney than the other tissues with all the viruses, ranging from several fold to 100-fold in excess. Higher levels of SV40 DNA copies were usually detected with the miRNA-negative mutants than with the WT viruses in the liver and kidney samples. This difference was observed despite the fact that fewer viral genome copies were inoculated per 10^7^ PFU with the mutant viruses than with the WT strains (Materials and Methods). There was a trend for viral loads of WT viruses to increase between days 7 and 14 in liver and kidney, in contrast to decreases in viral loads of the miRNA mutant viruses in most cases. Subsequent detailed analyses focused on liver and kidney specimens.

**Table 2:** Viral loads in different hamster tissues during early stages of infection by SV40.

Virus	Encode SV40 microRNA	Days p.i.	Average no. SV40 DNA copies/10^4^ cells^a^^,^ b ^,^ c				
			Liver	Kidney	Spleen	Lung	Brain
776-WT	+	7	114	134	26	13	Und
		14	299	597	7	4	Und
776-SM1	0	7	1352	235	13	8	209
		14	781	250	9	6	Und
SVCPC-WT	+	7	206	37	36	37	Und
		14	1403	117	20	9	Und
SVCPC-SM2	0	7	1971	437	64	103	6
		14	1687	231	19	Und	16

p.i., postinoculation; SV40, simian virus 40; Und, undetectable.

a Numbers represent the average of observed (nonadjusted) results from the tissues analyzed at each time point. See Table 1 for the number of tissues from different animals tested per virus at each time point (usually n = 4). Two or three separate fragments were assayed and the values averaged from each tissue from each animal (see Materials and Methods).

b The inoculum for each virus was 10^7^ plaque-forming units (PFU) per animal by the intracardiac route. The number of SV40 genome DNA copies per PFU varied for each viral stock: 776-WT, 1700; 776-SM1, 130; SVCPC-WT, 1800; SVCPC-SM2, 590. This resulted in the following ratios of viral genome copies inoculated: 776-WT:776-SM1, 13; SVCPC-WT:SVCPC-SM2, 3.

c Note that higher viral DNA copy numbers were more often detected in liver and kidney samples from animals exposed to microRNA-negative viruses than in those infected with wild-type viruses, even though fewer viral genome copies were inoculated with the mutants than with the wild-type viruses. This pattern held for most specimen collections through day 45 p.i.

### Persistence of viral DNA in hamster kidney following SV40 infections

Analysis of viral load data over time quantitated the preferential persistence of SV40 in the kidney compared to the liver and spleen. Virus levels, expressed as a ratio of day 45 to day 3 values for each virus (SV40 DNA copies/10^4^ cells), are shown (Table 3). Between 0.41 and 1.29 of day 3 levels were detected at day 45 in kidneys (equivalent to 41% and 129% of day 3 levels), in contrast to ratios of 0.10 to 0.17 in livers and even smaller ratios (≤0.10) in spleen. These day 45∶day 3 ratios show a higher relative persistence of SV40 in kidney than in liver by day 45 p.i. with all four viruses. The ratios of viral DNA levels in kidney∶liver and in kidney∶spleen at each time point are detailed in supplemental information (Table S1). These data suggest there was a reproducible trend for long-term viral persistence in the kidney, consistent with the idea that the kidney is the major reservoir for persistent infections in natural hosts by the well-characterized polyomaviruses. However, the retention of detectable viral DNA in the liver suggests that cells in the liver may be an unrecognized site of persistent infection by SV40 (Table 3). We noted there was a higher viral load ratio for 776-WT virus in kidney relative to SVCPC-WT virus (approximately 1.5-fold higher) as well as a higher ratio for the two miRNA mutants in kidney compared to the respective parental WT virus (about 2-fold higher) (Table 3). These results suggest that both a complex RR and the absence of viral miRNA can lead to higher chronic levels of SV40 viral DNA in the kidney of susceptible hosts. Virus was frequently detected in the kidney at day 270, ranging from 62% to 100% of animals tested (Table 1). However, only occasional animals (0–43%) had detectable virus remaining in the liver at day 270, and it is noteworthy that the 776 miRNA mutant (776-SM1) was not detected.

**Table 3:** Preferential persistence of SV40 in kidney compared to liver and spleen.

Virus	Encode SV40 microRNA	Ratio of day 45∶day 3 viral loads (SV40 DNA copies/10^4^ cells)^a^		
		Kidney	Liver	Spleen
776-WT	+	0.65	0.16	–
776-SM1	0	1.29	0.12	0.10
SVCPC-WT	+	0.41	0.10	0.05
SVCPC-SM2	0	0.73	0.17	0.01

SV40, simian virus 40.

a Values represent the ratio of the average observed (nonadjusted) results from day 45 and day 3 for each virus in the indicated tissues. The numbers of tissues from different animals tested per virus at each time point (usually n = 4) are listed (Table 1). Note the higher level of persistence of each virus in the kidney as compared to liver and spleen.

### Expression of SV40 miRNA in hamster tissues

The expression of the SV40 miRNA in hamster liver and kidney tissues was analyzed as described in Materials and Methods. Briefly, total RNA was harvested and then size-fractionated to enrich for small RNAs. The presence of the SV40 3p miRNA was determined via RT-PCR. Specimens were coded and tested without knowledge of sample identity. A sample was scored positive if its C_T_ was ≤35, which is within the linear range of this assay (as determined using a standard dilution series).

Expression of SV40 miRNA was detected in tissue samples from animals infected with WT SV40 strains, but not in tissues infected with miRNA-negative mutants (Table 4). Compared to cultured African green monkey BSC40 cells infected with SV40 at a high multiplicity of infection (MOI) (C_T_ 12.2), or to an abundant host miRNA in BSC40 cells (miR-Let7a, C_T_ 24.6), the levels of the SV40 miRNA in hamsters were lower (C_T_ 31–<35); likely reflective of the fact that only a small fraction of the assayed cells was infected. Twelve of 15 (80%) WT-infected samples tested positive for SV40 miRNA, including 7 of 7 (100%) kidney specimens and 5 of 8 (62%) liver samples. All control samples tested were negative for SV40 miRNA (0/6, 0%), including 4 miRNA mutant virus-infected tissues and 2 samples from uninfected hamsters. These results represent the first detection of SV40 miRNA expression *in vivo* in intact animals.

**Table 4:** Detection of SV40 miRNA in hamster tissues^a^.

Virus	Days p.i.	Tissue	
		Liver	Kidney
776-WT	3		++
	7	+	
	14	0	++
	28		++
	45	0	++
776-SM1	14	0	0
SVCPC-WT	3	+, 0	
	14	+, ++	++, ++
	28	+	++
SVCPC-SM2	3	0	
	14	0	
Control	8	0	
	270		0

a ++, +, 0 = detection of SV40 miRNA with C_T_35 as cut-off. ++ = ≤32 cycles; + = >32 to <35 cycles; 0 = ≥35 cycles. Each data point represents results for tissue from one animal. Two results shown for a given type of sample reflect independent results from two different animals.

### SV40 miRNA effects on infection profiles in hamster liver and kidney

**Figure 2:**
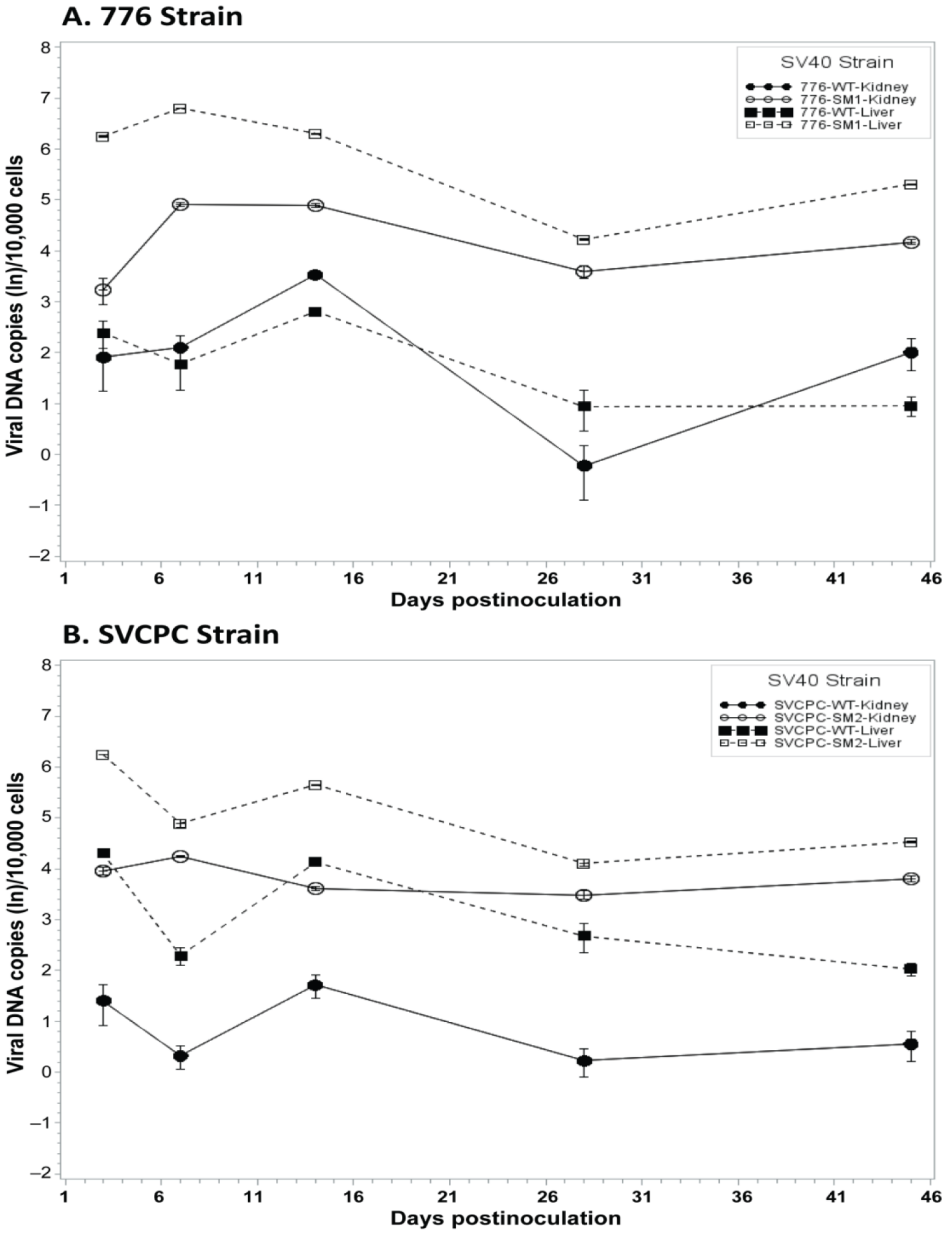
Effect of simian virus 40 microRNA on viral loads in hamster liver and kidney over time. (A) Strain 776, wild-type virus and microRNA-negative mutant SM1. (B) Strain SVCPC, wild-type virus and microRNA-negative mutant SM2. Viral loads are presented as the geometric mean titers of normalized (adjusted) viral DNA copies (natural log,) per 10,000 cells. The number of different animals analyzed per virus at each time point (usually n = 4) is shown in Table 1. Viral DNA levels were higher for the two microRNA mutants than for wild-type viruses in both liver and kidney through day 45. The differences between WT and mutant viruses were statistically significant (*p*≤0.05) for both viral systems in both tissues (see text for details). Error bars represent the standard deviation.

SV40 miRNA effects on the dynamics of SV40 infections in hamsters were then examined in detail. To directly compare the infection patterns of paired WT and miRNA-negative mutant viruses, observed viral genome counts were normalized as described in Materials and Methods, geometric mean titers were calculated, and expressed as viral DNA copies (ln) per 10,000 cells (Figure 2). In the 776 system, the viral loads for the SM1 mutant were consistently higher than those of the WT virus in both liver and kidney samples from day 3 through day 45 (Figure 2A). These differences between the two viruses were statistically significant at *p*<0.05 in both tissues at each time point, except for day 3 in the kidney. Similarly in the SVCPC system, viral DNA levels for the SM2 miRNA-negative mutant were higher than those of the WT virus in both liver and kidney from day 3 through day 45 (Figure 2B). The differences between these two viruses in both tissues were also statistically significant (*p*<0.05) at each time point, except for day 7 in the liver.

Viral DNA copies remained detectable at low levels at day 270 in most kidney specimens and less frequently in liver samples (Tables 1, 5). In contrast to the SVCPC-SM2 miRNA mutant, the 776-SM1 mutant was not detected in the liver of any hamsters tested at this late time point, suggesting that the SM1 infections may have been cleared from the liver. The differences in viral DNA loads at day 270 were statistically significant between the miRNA mutant and its WT virus in the SVCPC system in both tissues and between the two miRNA mutants in both tissues (Table 5). The difference in viral loads between the two WT viruses in the liver was also statistically significant.

**Table 5:** Persistent infections at day 270 in hamster liver and kidney by SV40 wild-type strains and microRNA mutants.

Virus	Encode SV40 microRNA	Day 270 viral loads (SV40 DNA copies/10^6^ cells)^a^^,^ b	
		Liver	Kidney
776-WT	+	11	181
776-SM1	0	Und	385
SVCPC-WT	+	12	210
SVCPC-SM2	0	22	1116

SV40, simian virus 40; Und, undetectable.

a Values represent the average of normalized viral DNA copies/10^6^ cells. The numbers of animals tested (6–9 each per virus and tissue) and those found to be virus-positive (0–8 each) are shown in Table 1.

b The following comparisons were statistically significant by the Wilcoxon rank-sum test at *p*≤0.05: SVCPC-SM2 vs. SVCPC-WT in both liver and kidney, SVCPC-SM2 vs. 776-SM1 in both liver and kidney, and 776-WT vs. 776-SM1 in liver.

### Effect of SV40 miRNA and RR structure on relative viral levels in hamster tissues

**Figure 3:**
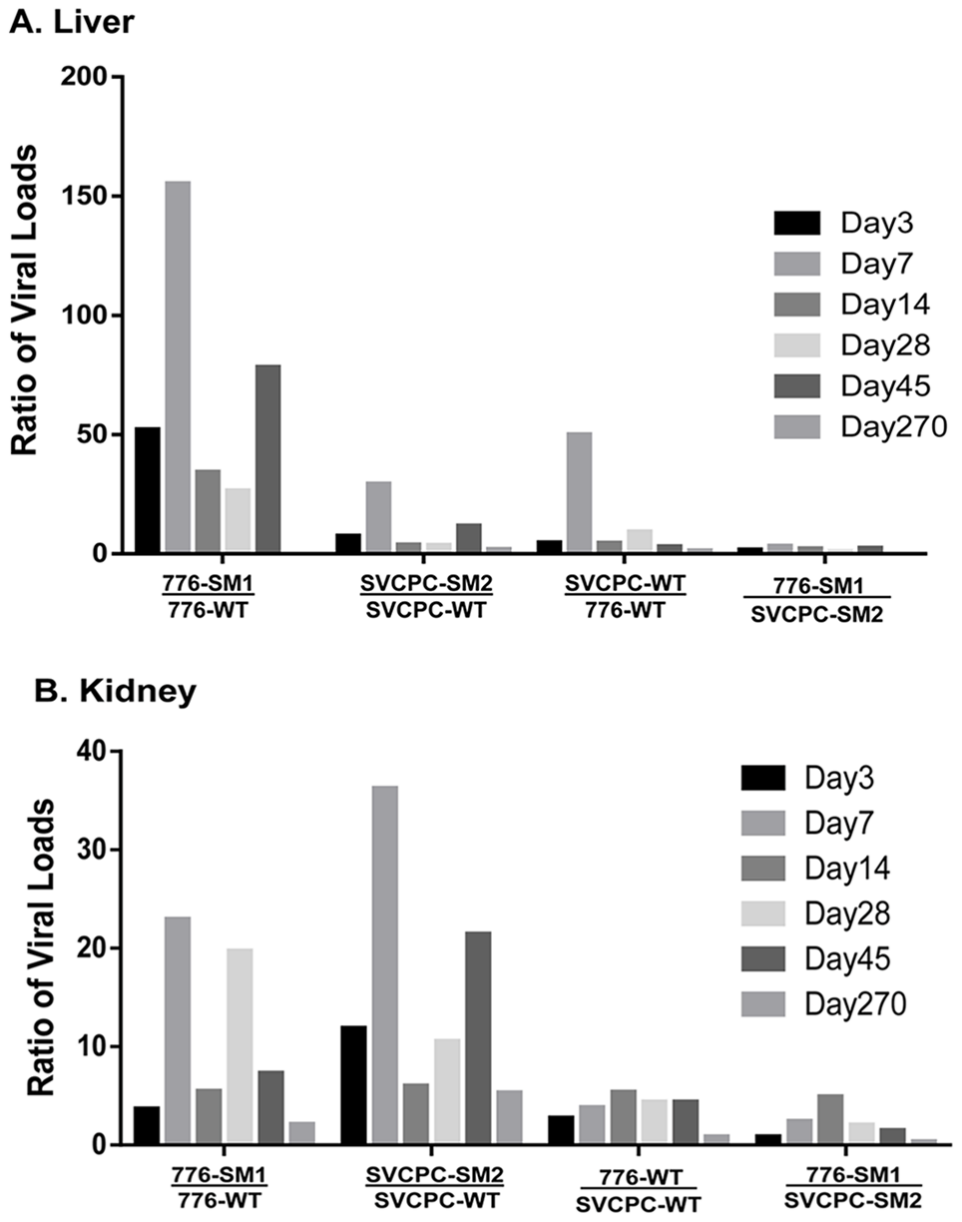
Effects of simian virus 40 microRNA and regulatory region structure on relative viral loads in hamster tissues over time. (A) Liver. (B) Kidney. Bars represent ratios of viral loads for the comparisons listed below each set. Individual bars within each set reflect days 3 to 270. Ratios were calculated using means of normalized viral load data. (No bars are shown for day 270 in the liver for the two ratios involving 776-SM1 as no virus was detected in the livers of infected animals at that time point.) Numbers of animals analyzed are shown in Table 1. Note the marked increases of the microRNA mutants compared to wild-type viruses in both tissues.

The relative influence of the structure of the SV40 RR and of the presence or absence of viral miRNA on the dynamics of virus infection in both liver and kidney was next evaluated (Figure 3). The bars represent the ratios of the means of normalized viral load titers between the two viral strains listed below each set; individual bars within a set reflect days 3 to 270 p.i. The two miRNA-negative mutants had relatively higher viral DNA loads than the matched parental WT viruses in the liver, reaching as much as >100-fold higher (Figure 3A). No bars are shown for day 270 in the liver for the two comparisons involving the 776-SM1 mutant because 776-SM1 viral DNA was not detected in the livers of infected animals at day 270 (Tables 1, 5). The effect in the liver of the absence of miRNA was more dramatic with the 776 system than with SVCPC. The mutant viruses also displayed higher viral DNA loads than the parental WT viruses in kidneys, differences that reached >20-fold (Figure 3B). The WT and mutant viruses with complex RR (776-WT, 776-SM1) reached higher viral loads (≤5-fold) in kidney as compared to the viruses with simple RR (SVCPC-WT, SVCPC-SM2). The loss of viral miRNAs had a larger impact than the structure of the viral RR on the amount of viral DNA in infected tissues.

### Viral gene expression in liver and kidney tissues

Expression levels of SV40 early (T-ag) and late (VP1) regions in liver and kidney were determined as described in Materials and Methods. The single copy hamster vimentin gene was used to normalize viral transcript levels; results are presented as SV40 mRNA copies per 10^6^ vimentin mRNA molecules (Table 6). The viral early region was expressed at higher levels than the late region in both liver and kidney samples at time points ranging from 3 to 45 days p.i. The WT viruses and miRNA-negative mutants displayed similar expression patterns, with transcript numbers routinely higher in liver than in kidney. It was noted that the T-ag mRNA levels from 776-SM1 consistently exceeded those of the 776-WT virus in the liver. There appeared to be a trend for T-ag transcript levels in the liver to decrease over time, whereas those in the kidney increased.

**Table 6:** SV40 mRNA expression in hamster liver and kidney tissues.

Virus	Encode SV40 microRNA	Days p.i.	mRNA copies/10^6^ vimentin copies^a^			
			T-ag (early)		VP1 (late)	
			Liver	Kidney	Liver	Kidney
776-2E	+	3	2784	Und	186	Und
		7	1673	216	118	Und
		45	484	135	Und	Und
776-SM1	0	3	9329	Und	319	Und
		7	2681	63	129	Und
		45	7337	67	163	Und
SVCPC-WT	+	3	8708	71	476	Und
		14	19,628	38	651	Und
		45	2331	147	Und	34
SVCPC-SM2	0	3	1851	59	239	Und
		14	15,418	179	903	19
		45	4450	237	229	38

SV40, simian virus 40; p.i., postinoculation; Und, undetectable.

a Numbers represent the average of observed viral transcript results from the tissues from two different animals analyzed at each time point. The relative expression of vimentin mRNA copies (used to normalize viral expression) was about 3-fold higher in kidney samples as compared to those from liver.

### Virus isolation attempts

Recovery of infectious virus was attempted from liver and kidney specimens from hamsters exposed to the four virus strains. Tissue lysates were prepared and tested as described in Materials and Methods. Tissues included kidney samples from animals sacrificed at 7, 14, 45 and 270 days p.i. and liver samples from animals harvested at 7 and 14 days p.i. (2 or 3 animals each). Infectious virus was detected after passage of kidney specimens collected on day 7 (776-SM1, 3 of 3; SVCPC-SM2, 3 of 3; and SVCPC-WT, 1 of 2). Other kidney samples were negative and no infectious virus was recovered from liver materials tested. These results suggest that the majority of the viral DNA detected in these tissues was not present in the form of infectious virus particles by 14 days p.i.

### Lack of effect of SV40 miRNA on cell transformation *in vitro*

Focus-forming assays using primary mouse embryo fibroblasts were carried out as described in Materials and Methods to determine if the absence of SV40 miRNA affected transformation by the virus *in vitro*. Three sets of WT and miRNA-negative mutant viruses were tested, including the 776 and SVCPC strains. Also tested in the transformation studies were SVCPC-based recombinants carrying either the 776-WT large T-ag gene (SVCPC-776-WT) or the T-ag gene with the SM1 mutation (SVCPC-776-SM1). Replicate infected cultures were harvested and transformed foci counted at 3 and 5 weeks p.i. For each matched pair of viruses, numbers of foci were expressed as the percentage of WT virus transformed foci at 5 weeks (set as 100%). All six viruses were able to transform mouse cells *in vitro* (Table 7). The foci induced by the mutant viruses were somewhat slower growing and appeared later, but by 5 weeks p.i. there was an insignificant 2-fold difference in the relative number of foci produced by the 776-derived mutants. The SVCPC-WT transformed foci were slower at appearing than those induced by the 776-WT viruses, and the SVCPC-SM2-induced foci had a slower growth rate. Results showed that the lack of miRNA neither enhanced nor abolished the transforming ability of SV40 in cultured mouse cells.

**Table 7:** Transformation of primary mouse embryo fibroblasts by SV40 parental and microRNA-negative viruses.

Virus	Encode SV40 microRNA	Relative no. of transformed foci (%)^a^	
		3 weeks	5 weeks
776-WT	+	76	100
776-SM1	0	12	48
SVCPC-776-WT	+	55	100
SVCPC-776-SM1	0	20	53
SVCPC-WT	+	21	100
SVCPC-SM2	0	3	16

SV40, simian virus 40.

a Replicate plates (n = 4–6) were harvested per virus at each time point. Numbers of transformed foci per 1×10^5^ cells infected by each virus were normalized to the value for the WT strain of each pair of viruses at 5 weeks, which was set as 100%. The overall transformation frequency was ∼0.05%.

### Lack of effect of SV40 miRNA on tumor development in hamsters *in vivo*

The WT and miRNA mutants were tested for tumor induction in hamsters to determine if miRNA effects were manifest during *in vivo* oncogenesis. Weanling (21-day-old) animals were inoculated intraperitoneally with 1×10^7^ PFU of virus and observed for 12 months. A third pair of viruses (SVCPC-776-WT and SVCPC-776-SM1) was included in the *in vivo* tumorigenicity experiment. All six viruses induced tumor formation. There was no significant difference in tumor incidence between a given WT virus and its miRNA-negative mutant for any of the three virus pairs (Table 8). There was also no difference in the latency period (time to tumors) between the WT and miRNA mutant viruses. As observed in earlier studies, virus strains with a simple RR (SVCPC) were more oncogenic than virus strains with a complex RR (776) [16]. Susceptibility to tumor induction by SV40 is age-related in hamsters. Tumors were not observed in the animals inoculated by the intracardiac route in this study because those animals were older at the time of virus exposure (Materials and Methods).

**Table 8:** Lack of effect of SV40 microRNA on tumor development in Syrian golden hamsters following intraperitoneal inoculation.

Virus	Encode SV40 microRNA	No. tumors/no. animals (%)	*p* value^a^	Time to tumors, weeks Median (range)	*p* value^a^
776-WT	+	2/20 (10)	0.27	34 (29–39)	0.67
776-SM1	0	1/20 (5)		34	
SVCPC-776-WT	+	8/13 (62)	0.14	28 (21–33)	0.26
SVCPC-776-SM1	0	10/20 (50)		33 (24–51)	
SVCPC-WT	+	4/10 (40)	0.50	26 (21–43)	0.83
SVCPC-SM2	0	8/20 (40)		33 (21–49)	
Control cell lysate	0	0/10 (0)		–	

SV40, simian virus 40.

a *p* values for comparisons of % tumors and of time to tumors were determined by the Z test and the proportional hazards regression test, respectively.

**Figure 4:**
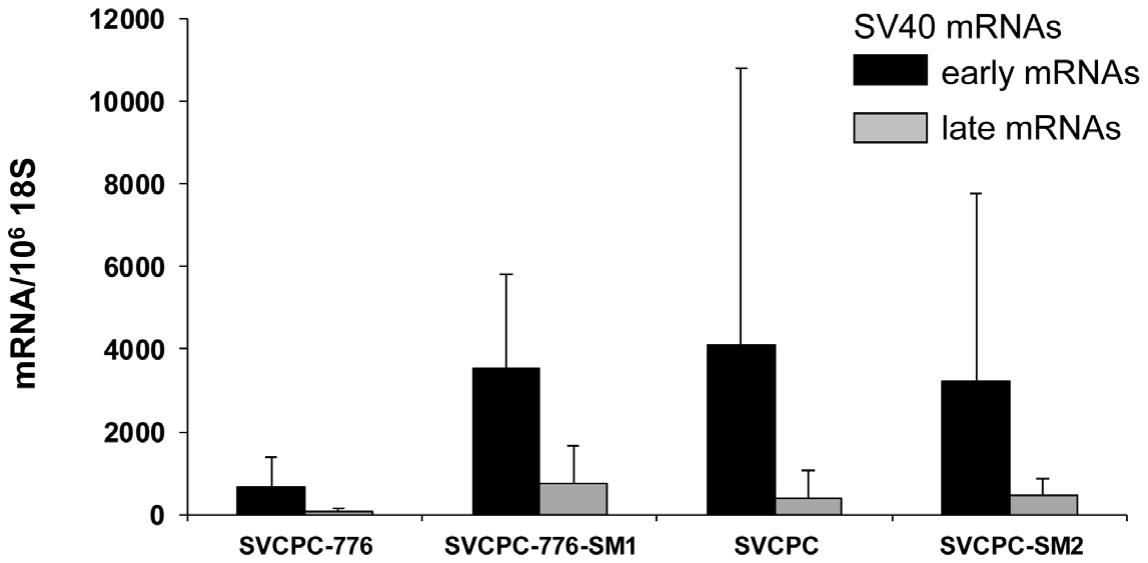
Detection of simian virus 40 (SV40) early and late mRNAs in hamster tumors induced by wild-type viruses and microRNA-negative mutants following intraperitoneal inoculation. SV40 transcripts in hamster tumors induced by wild-type and microRNA-negative viral strains were reverse transcribed, quantitated by real-time quantitative polymerase chain reaction and expressed as the average number of SV40 mRNA copies per 10^6^ copies of 18S ribosomal RNA. The numbers of tumors analyzed for each viral system were the following: SVCPC-776 = 5, SVCPC-776-SM1 = 5, SVCPC = 3, and SVCPC-SM2 = 4. The error bars represent the standard deviation.

Tumors induced by WT and mutant viruses were characterized for the content of SV40 DNA and for viral gene expression. The following tumor-associated SV40 DNA levels were determined by RQ-PCR [copies/cell, mean (range)]: SVCPC-776 = 32 (1–50) [5 tumors], SVCPC-776-SM1 = 14 (1–48) [5 tumors]; SVCPC = 15 (1–23) [3 tumors], and SVCPC-SM2 = 10 (6–14) [4 tumors]. Tests were not carried out to determine the integrated or episomal status of those genome copies in the tumor cells. Total RNA was isolated from the same tumors, reverse transcribed, and SV40 transcripts quantitated by RQ-PCR (Figure 4). Results are expressed as the number of SV40 transcripts per 10^6^ copies of 18S ribosomal RNA (GenBank accession No. X03205.1). Both early and late viral mRNAs were detected in the tumors, with the SV40 early mRNAs being more abundant than the late mRNAs.

Antibody responses to SV40 T-ag as well as neutralizing antibody responses to SV40 were measured as described in Materials and Methods (Table 9). All but one of the 33 tumor-bearing animals (97%) produced T-antibodies with similar median titers. Similarly, all (100%) of the tumor-bearing animals produced SV40 neutralizing antibody with comparable neutralization titers, regardless of the virus strain inoculated. Results were more variable among the 70 animals that failed to develop tumors. The frequency of T-antibody responses in tumor-free animals ranged from 0% to 72% per group with median titers that tended to be lower than those in animals with tumors. Animals exposed to the 2E viruses (776) tended to produce T-antibody more frequently than those exposed to the 1E (SVCPC) viruses. There were no discernible differences in patterns of T-antibody responses in tumor-free animals related to the inoculation of miRNA-positive or -negative viruses. Those responses indicate there was sustained T-ag expression in the animals adequate to induce the production of stable T-antibodies. From 42% to 80% of a given group produced neutralizing antibody with median titers that tended to be lower than those in the tumor-bearing animals in the same group.

**Table 9:** Antibody responses in tumor-bearing and tumor-free hamsters inoculated intraperitoneally with wild-type SV40 and microRNA-negative virus mutants.

Virus	Encode SV40 microRNA	Tumor bearing	SV40 T-antibody		SV40 neutralizing antibody	
			No. pos./No. tested (%)	Titer Median (range)	No. pos./no. tested (%)	Titer Median (range)
776-WT	+	+	2/2 (100)	100/1000	2/2 (100)	2000 (2000)
		0^a^	13/18 (72)	100 (10–1000)	13/18 (72)	200 (20–2000)
776-SM1	0	+	1/1 (100)	1000	1/1 (100)	2000
		0	12/19 (63)	100 (5–1000)	12/19 (63)	200 (20–2000)
SVCPC-776	+	+	8/8 (100)	1000 (100–1000)	8/8 (100)	10,000 (2000–100,000)
		0^a^	0/5 (0)	–	3/5 (60)	200 (20–200)
SVCPC-776-SM1	0	+	9/10 (90)	100 (10–5000)	10/10 (100)	2000 (200–100,000)
		0	7/10 (70)	10 (10–100)	8/10 (80)	20 (20–200)
SVCPC-WT	+	+	4/4 (100)	100 (100–5000)	4/4 (100)	200 (200–10,000)
		0	3/6 (50)	10 (10)	4/6 (67)	20 (20–200)
SVCPC-SM2	0	+	8/8 (100)	100 (100–5000)	8/8 (100)	200 (200–40,000)
		0	3/12 (25)	100 (10–100)	5/12 (42)	20 (20–200)
Controls (no virus)	0	0	0/10 (0)	—	0/10 (0)	—

SV40, simian virus 40.

a From reference [10].

## Discussion

One goal of this study was to identify tissues susceptible to SV40 infection and those that became persistently infected. Hamster liver, kidney, and spleen samples were frequently viral DNA positive through day 45 p.i.; lung and brain tissues were less often virus positive and muscle specimens were consistently virus negative (Table 1). Higher viral loads (SV40 DNA copies/10^4^ cells) were routinely detected in liver and kidney samples as compared to other tissues, with virus titers in the liver often exceeding those in the kidney during the early phases of infection (Table 2, Figure 1, Figure 2). These results show that tissues varied in their susceptibility to infection by SV40. Both SV40 miRNA in WT infections and viral transcripts were detected in liver and kidney samples (Tables 4 and 6), evidence of viral gene expression in infected cells. Most kidney samples were virus-positive through day 270, whereas liver tissues remained infected in most animals for as long as 45 days p.i. and in some animals as late as day 270. It is possible that livers are an unrecognized site of polyomavirus persistence and it will be important in future studies to identify the target cells in the liver that support long-term SV40 infections. Evidence suggests that polyomaviruses can be transmitted by the fecal/urine–oral route and can be found in lymphoid-rich samples from hamsters and humans, so cells in the gastrointestinal tract and in lymphoid tissues are of interest as well [2], [12], [14], [21]–[26] (Tables 1, 2). Results from an earlier study of SV40 vertical transmission in hamsters are compatible with the tissue susceptibilities described here [15]. In that investigation pregnant hamsters were inoculated intraperitoneally with 1×10^7^ PFU of SV40 strains and were sacrificed at different times up to 24 days p.i. Kidney and spleen were infected in all 16 maternal animals, and lung in some animals, confirming that cells in these tissues are susceptible to infection by SV40.

Viral miRNAs have been discovered for several virus families, including the herpesviruses and the polyomaviruses [19], [20], [27]–[33]. The functions of many of the viral miRNAs have not yet been determined but are proposed to be involved in prolonging the viability of infected cells and in promoting immune evasion to increase virus survival [19], [30], [31], [34]. Viral miRNA effects are likely to be of significance to the establishment, maintenance, and/or reactivation of persistent infections in susceptible hosts by viruses that commonly establish chronic infections. Very few studies have examined the effect of viral encoded miRNAs on viral infection in an intact host. This study provides the first evidence to our knowledge of the detectable expression and functional effects of SV40 miRNAs on polyomavirus SV40 acute and persistent infections *in vivo* in a susceptible host. Hamsters infected with SV40 mutants unable to express the viral miRNAs frequently contained higher levels of viral DNA in liver and kidney tissues than animals exposed to the WT viruses. This finding is compatible with the original report that SV40 miRNAs reduce the expression of SV40 T-ag in infected cells [20], as higher levels of T-ag in mutant-infected cells could be expected to support enhanced viral DNA replication.

SV40 strains tested in this study differed in the structures of their RR. Variants with a duplication of the enhancer element in the RR (i.e., complex RR) can replicate to somewhat higher levels (∼2-fold) in DNA replication assays and in cell cultures than variants with a simple RR [35], [36]. It has also been shown that overexpression of T-ag stimulates replication of human JCV and BKV isolates with simple RR *in vitro* [37]. In the current study, the SV40 776 strains with complex RR exhibited approximately 2- to 5-fold higher viral loads in hamster kidney than the SVCPC strains with simple RR (Figure 3B), suggesting an RR effect on virus replication *in vivo* in kidney cells. A complex RR would likely have a more pronounced enhancement on virus replication in immunocompromised hosts. It has been observed that natural SV40 variants with rearranged RR arise *de novo* in infected immunocompromised rhesus monkeys [38] and this same phenomenon has been observed with human polyomaviruses. JCV isolates with a simple RR are shed in the urine, whereas isolates with a complex RR are recovered from the brain and cerebrospinal fluid of patients with progressive multifocal leukoencephalopathy [39]–[41]. The emergence of BKV with a rearranged RR is linked to increased viral replication and increased BKV-associated nephropathy disease in kidney transplant recipients [42].

Notably, our results showed that the loss of viral miRNA had a greater effect on viral DNA loads than that of the complex RR structure. However, the absence of viral miRNA resulted in a relatively larger increase of viral DNA in the 776 system than it did with the SVCPC strain (Figure 3). We speculate that this stronger effect might reflect the combination of the complex viral RR and the absence of viral miRNA. Another virus distinction is that there are several nucleotide differences between strains 776-WT and SVCPC-WT in the T-ag gene, plus a 9-bp insertion in the SVCPC gene, changes which result in 6 amino acid differences between the two T-ags (Figure S1) [43], [44]. However, we do not believe these changes affected the level of viral DNA in hamster tissues. We compared the average titers of virus stocks of 776-WT (n = 18) and SVCPC-WT (n = 15) prepared in our laboratory at different times over the last decade. There was no statistical difference (p>0.05) between the groups, indicating no obvious differential T-ag effects on virus replication.

SV40 miRNA was detected (Table 4) when levels of SV40 late transcripts were low (Table 6) and infectious virus was not recovered. This raises the question of how functional SV40 miRNAs were expressed in the absence of abundant lytic infections of hamster cells. The most likely explanation is that some cells in a given tissue were supporting complete cycles of virus replication and the late transcription in those cells produced adequate amounts of miRNA to yield the observed results. Alternatively, it has been speculated there may be a mechanism to produce miRNAs in polyomavirus chronically infected cells that is not dependent on late viral gene expression [19]. Importantly, it has been shown recently that BKV miRNA is expressed in BKV-infected cultured cells before the onset of viral DNA replication [45]. The BKV study observed increased replication of miRNA-null mutants with an archetypal RR in renal proximal tubule epithelial cells, similar to our findings with SV40 in hamsters. However, the absence of BKV miRNA had no stimulatory effect on replication of BKV variants with a rearranged RR, in contrast to our SV40 results [45]. Whether this reflects a fundamental difference between BKV and SV40, the influence of cell culture *vs. in vivo* conditions, or some other experimental variable is a topic for future study.

It has been proposed that SV40 miRNA may function to mediate evasion of the host response to infected cells [20]. However, in this study the mutant viral loads were not reduced more quickly than those of WT viruses over the first 45 days, suggesting that mutant virus-infected cells were not cleared more quickly by the host immune response (Figure 2). There was a statistically significant difference between 776-WT and 776-SM1 in the number of animals that retained viral DNA in the liver at day 270, but not between SVCPC-WT and SVCPC-SM2. There are several possible explanations for the inability to detect pronounced miRNA effects on viral clearance. One possibility is that the original model does not encompass the most important role for the miRNA during infections. Perhaps SV40 has an unrecognized non-miRNA mechanism for interfering with the host immune response to allow establishment of chronic infections, making the presence or absence of viral miRNAs irrelevant. Another possibility is that the SV40 miRNAs may have been unable to target a cellular mRNA critical to the host response because of sequence mismatches between the primate virus and the hamster genome. (It has been proposed that human JCV and BKV miRNAs target a stress-induced human cell ligand recognized by natural killer cells to mediate killing of virus-infected cells [46].) Alternatively, the Syrian golden hamster immune system may possess some unusual characteristic that makes the animals prone to establishment of persistent infections by microbial agents, thereby over-riding or negating possible miRNA effects. For example, hamsters are known to be susceptible to infections by numerous viruses and a parasite able to infect humans [47]–[56]. Finally, although viral miRNA expression was detected and differences were observed in viral loads between the WT and viral miRNA mutant viruses in hamster tissues, perhaps the levels of the viral miRNAs were insufficient to mediate effects on viral clearance. Because of these unknown factors, we cannot conclude that the viral miRNA never affects the host immune response. It is noteworthy that our findings are similar to the observation that a miRNA-negative mutant of murine polyomavirus generally behaved like the WT virus in experimentally infected mice with respect to viral clearance and cellular immunity. Although expression of miRNAs was not directly demonstrated, the genome copy loads of the mutant tended to exceed those of the WT polyomavirus in adult mouse kidneys [29].

There are several limitations to this study. Hamsters are not the natural host for SV40, but they represent one of the few systems that can be used for investigations of polyomavirus infections in intact animals. The intracardiac route of inoculation that was used to allow determination of the breadth of susceptible tissues and the sites of viral persistence was not the natural route of infection. It was beyond the scope of this study to compare different routes of inoculation. In addition, the dose of virus inoculated was high compared to the low level of virus encountered in a natural infection. It is conceivable that the nonnatural route of inoculation coupled with the high level of inoculum might have masked possible phenotypes. The virus did not appear to produce infectious progeny beyond the early stages of infection, although it is possible that additional tests of more tissue samples might have detected extended replication. Alternatively, it is possible that SV40 enters a persistent, nonproductive state relatively soon after infection.

The study raised several challenging subjects for follow-up investigations. These include a potential role of viral miRNA in reactivation of SV40 persistent infections, the molecular basis for the difference between viral miRNA inhibitory effects on BKV and SV40 viruses with complex RR, the mechanism of expression of SV40 miRNA in the absence of abundant viral replication, and the interaction of SV40 with susceptible cells in the liver.

In summary, this study demonstrated for the first time that SV40 miRNAs are expressed and are functional *in vivo* in infected hamsters. We showed that SV40 has a broad tissue tropism, identified tissues that support viral DNA replication (kidney, liver, spleen, lung and brain), and found that kidneys are the major site for long-term persistence with livers a possible secondary site. One role for the miRNA appears to be to reduce viral loads in infected tissues. We also showed that viral miRNA does not appear to affect SV40 tumorigenicity in hamsters, the traditional assay for SV40 oncogenic potential.

## Materials and Methods

### Ethics statement

Outbred Syrian golden hamsters (*M. auratus*) were obtained from Harlan Laboratories and were housed in the biohazard facility in the Center for Comparative Medicine at Baylor College of Medicine. The animals were maintained in accordance with established national guidelines as outlined in the Guide for the Care and Use of Animals [57] and the Animal Welfare Act. The Center for Comparative Medicine is fully accredited by the Association for Assessment and Accreditation of Laboratory Animal Care International (Animal Welfare Assurance Number A3823-01). The studies were approved (Protocol Number AN668) by the Institutional Animal Care and Use Committee of Baylor College of Medicine.

### Animal experiments

Procedures for intracardiac and intraperitoneal injections have been described [10], [16]. We have excellent survival outcomes (≥99%) with both routes of inoculation. Five- to 7-week-old female hamsters were used for intracardiac inoculations for the pathogenesis studies, whereas 3-week-old male and female hamsters were employed for intraperitoneal injections for the tumorigenicity studies. Animals were inoculated with 1×10^7^ PFU of virus in 0.1 ml by the intracardiac route or 1×10^7^ PFU of virus in 0.5 ml intraperitoneally. Control animals were inoculated with uninfected cell lysate under the same conditions. Animals were sacrificed at designated days p.i., at evidence of neoplasia or debility, or at the termination of experiments. Euthanasia involved isoflurane overdose and exsanguination by cardiac puncture. Necropsies were carried out on all the animals inoculated by the intracardiac route and some of those inoculated by the intraperitoneal route. Tissue samples were collected and were stored frozen or preserved in RNAlater.

### Viruses

Two natural SV40 strains from different phylogenetic groups were studied [43]. Reference strain 776 (complex RR) was isolated from an adenovirus type 1 vaccine seed stock [58]. SVCPC (simple RR) was recovered from a pediatric brain tumor [59]; the same virus has been detected in other human cancers [21], [59]–[61] and in the Russian oral poliovaccine [62]. The structures of the 776 and SVCPC viral RR are shown in reference [15]. The miRNA-negative mutant derived from 776-WT virus (776-SM1) was obtained from C. Sullivan [20]. The construction of recombinant virus SVCPC-776-WT was described previously [16]. This recombinant contains the RR, late region, the N-terminal part of T-ag, and the small t-ag coding region of strain SVCPC with the C-terminal part of T-ag (BstXI and BamHI restriction fragment) from 776-WT. Recombinant SVCPC-776-SM1 was constructed following the same strategy and contains the C-terminal T-ag fragment from 776-SM1.

Due to nucleotide polymorphisms in the miRNA region, the predicted sequence of the pre-miRNAs of SVCPC-WT and 776-WT differ slightly. Consequently, the amino-acid sequences of the T-ags of SVCPC-WT and 776-WT also slightly diverge. The miRNA-negative mutant derived from SVCPC-WT virus (SVCPC-SM2) was created by mutagenesis of 18 nucleotide positions in the pre-miRNA region analogous to that of 776-SM1. These mutations disrupt the hairpin structure of the SVCPC-WT pre-miRNA on the late strand while leaving intact the amino acid-coding potential of T-ag on the early strand (Figure S1) [20]. Three overlapping fragments covering the miRNA region were PCR-amplified with six mutagenic primers using SVCPC-WT or 776-SM1 DNA as a template. Three PCR primer sets were MA1 5′-TTCCTGGGGATCCAGACATGATAAG-3′ and MIRA2 5′-CTCCGCAGCCTTCGCAGTCCT-3′ on the SVCPC-WT template, MIRB1 5′-AGGCTGCGGAGCTTGAAACGAAC-3′ and MIRB2 5′-AGCCAGGAAAATGCTGATAAAAATG-3′ using the 776-SM1 template, and MIRC1 5′-AGCATTTTCCTGGCTGCTGTCATCATCA-3′ and SVPST 5′-AAAACACTGCAGGCCAGATTTG-3′ on the SVCPC-WT template. Three mutated fragments were subsequently joined together by overlapping PCR using outer primers. The resulting mutated fragment was cleaved with restriction enzymes PstI and BamHI and used to replace the corresponding part of the genome of SVCPC-WT also cleaved with the same restriction enzymes. The final miRNA-negative mutant virus was denoted SVCPC-SM2.

Each recombinant construct was sequenced to confirm that no accidental mutations had been introduced and was transfected into TC-7 cells to confirm its infectivity. The mutant viruses were shown to be defective for production of viral miRNAs using a test designed to detect early mRNA cleavage fragments that are generated by functional miRNA [20]. TC-7 cells were infected at a multiplicity of infection of 2 PFU/cell with each virus and total RNA was harvested at 65 hours p.i. using the RNAqueous-4 PCR kit (Ambion) and treated with DNase I. Total RNA was Northern blotted with a probe specific to the 3′ region of SV40 early mRNA (nucleotides 2681–2845, prepared by PCR), radiolabelled with α^32^PdCTP using the DECAprimeII kit (Ambion). The characteristic early mRNA 3′ cleavage fragment about 260-nt in size was generated by each of the three WT viruses (776-WT, SVCPC-776-WT, and SVCPC-WT) that produce intact miRNAs. In contrast, the miRNA-negative mutant viruses (776-SM1, SVCPC-776-SM1, and SVCPC-SM2) produced only full-length early transcripts without any detectable miRNA-generated 3′ cleavage fragment.

Virus stocks were prepared in TC-7 monkey kidney cells. Infectious virus titers were determined by plaque assay [63]. The numbers of viral genome copies in virus stocks were determined using an RQ-PCR assay.

### Nucleic acid extractions and PCR analysis

Small pieces of frozen tissues were minced and digested overnight with Proteinase K and nuclei lysis solution. Proteins were precipitated and removed and then the DNA was precipitated with isopropanol, washed, resuspended in Tris-EDTA buffer (pH 8.0), and stored at −20°C [16]. Two or three random fragments from each tissue sample were processed and assayed independently; those values were averaged to represent the results from that tissue.

SV40 DNA was detected and quantified by an RQ-PCR assay [64]. Five µl of DNA sample were assayed in a final reaction volume of 25 µl using 40 cycles of amplification. The single-copy hamster vimentin gene was measured by RQ-PCR to evaluate the quality of each DNA extract and to normalize viral gene copy numbers to cell numbers [15]. Vimentin copies per reaction were divided by 2 to determine cell equivalents per reaction. A sample was omitted from viral analysis if less than 10,000 cell equivalents were amplified in a vimentin reaction. For those samples adequate for further analysis, RQ-PCR reactions were considered virus positive if ≥10 viral genome copies per reaction were detected. Viral loads are expressed as SV40 DNA copies per 10^4^ cells. Total RNA was extracted from samples preserved in RNAlater (Ambion) using the RNAqueous-4 PCR kit (Ambion) with DNase 1 treatment. RNA was reverse transcribed as described and then quantitated by RQ-PCR [14]. As a control, vimentin RNA or 18S ribosomal RNA was amplified and used to normalize SV40 RNA. Data are expressed as mRNA copies per 10^6^ vimentin molecules (liver and kidney tissues) or copies per 10^6^ 18S molecules (hamster tumors). Primer and probe sequences have been reported [14].

The number of SV40 genome copies per PFU varied among the four virus stocks for unknown reasons: 776-WT, 1700 genome copies/PFU; 776-SM1, 130 genome copies/PFU; SVCPC-WT, 1800 genome copies/PFU; and SVCPC-SM2, 590 genome copies/PFU. To directly compare viral loads in hamster tissues between virus strains (based on PCR assays which detect viral DNA copies), adjustments were made to account for the different number of viral genome copies in each 1×10^7^ PFU inoculum given the hamsters. SV40 DNA levels determined by PCR for a given sample were normalized by dividing the number of observed SV40 DNA copies by the number of viral DNA copies (×10^−9^) inoculated for that virus (i.e., if a viral load value was “X” copies of SV40 DNA per 10^4^ cells for an SVCPC-WT sample, “X” would be adjusted by dividing by 18). Normalized values were used when the dynamics of infection were compared between different viruses. Observed (non-adjusted) viral load values were used for analyses involving a single virus strain.

### MicroRNA analysis

Portions of frozen livers and kidneys from infected or uninfected hamsters were coded and assayed without knowledge of sample identity. The samples were lysed in 1 mL of TRIzol reagent (Life Technologies) containing Lysing Matrix B tissue grinding tubes (MP Biomedicals) by subjecting the tubes to 3 cycles of a 1-minute duration of tissue grinding at the maximum speed using a Mini-Beadbeater-16 (Biospec Products) followed by 1 minute of cooling on ice. The lysed samples were transferred individually to an additional 4 mL of TRIzol followed by RNA isolation according to the manufacturer's standard protocol. To enrich for small RNA, gel fractionation was performed. Total RNA was first subjected to electrophoresis on a Tris-borate-EDTA-Urea-15% polyacrylamide gel. The gel portion between the bromophenol blue and xylene cyanol markers (approximately 10–35 nucleotides) was excised and then cut into smaller pieces. The gel pieces were soaked in 15 mL of 1 M sodium chloride solution to allow RNA elution overnight at 4°C, with gentle rocking. The eluted small RNA supernatant was transferred to a Vivaspin 15R 2000 MWCO centrifugal concentrator (BioExpress) and spun at 3000 *g* until the supernatant was concentrated down to approximately 500 µL. The small RNAs were precipitated overnight at −20°C in one-tenth volume of 3 M sodium acetate, pH 5.2, and an equal volume of isopropanol. The small RNA was reverse transcribed using the manufacturer's (Taqman, Life Technologies) primers (designed to recognize the SV40 3p miRNA) and the SuperScript III reverse transcriptase, using a modified version of the manufacturer's protocol: 16°C for 30 minutes, followed by 60 cycles of 30°C for 30 seconds, 42°C for 30 seconds, and 50°C for 1 second, and a final incubation at 85°C for 5 minutes. The reverse transcription product was used in the Taqman Small RNA assay for detection of the SV40 3p miRNA (Life Technologies) and the assay was performed on a ViiA 7 real-time PCR system (Life Technologies) according to the manufacturer's protocol. The linear range of the assay was determined using a dilution series of total RNA (from SV40 776-infected BSC-40 cells at an MOI of 10) as the standard. This analysis revealed linearity down to C_T_ 39. We also estimated the sensitivity of our assay. The total number of cells assayed was held constant at 300,000 while altering the ratio of infected (776- infected BSC-40, MOI of 10) to uninfected cells. This resulted in an estimate of sensitivity ranging between 1 in 8,000 and 1 in 100,000 infected to uninfected cells. To minimize the chances of false positives, a conservative cutoff of C_T_ 35 was chosen to score positive for miRNA detection. For comparison, we also calculated the SV40 3p miRNA and miR-Let7a (Taqman Life Technologies) levels in BSC40 African green monkey cells infected with SV40 (strain 776, MOI 10) and harvested RNA at 24 hours postinfection. The average of at least three independent technical replicates was used to calculate C_T_ for all analyses.

### Antibody assays

Antibody responses to SV40 T-ag were detected and titered by indirect immunofluorescence as described [10]. SV40 neutralizing antibodies were measured using a plaque-reduction assay in TC-7 cells [10].

### Virus transformation

Focus-forming assays to quantitate SV40 transformation used primary mouse embryo fibroblasts infected with different strains of SV40 at 5 PFU/cell [16]. Replicate plates (usually 4–6) were harvested at 3 and 5 weeks p.i. and the number of transformed foci determined. For each WT and mutant virus pair, numbers of transformed foci per 1×10^5^ cells infected were calculated as the percentage of WT foci at 5 weeks (set as 100%). The transforming frequencies overall were about 1 per 2000 infected cells.

### Virus rescue

Tissue fragments were minced, frozen and thawed two times, and the cell debris pelleted by centrifugation. The pellets were resuspended in 200 µl buffer and both the lysate supernatants and the lysate pellets were inoculated into TC-7 cell cultures. These cultures were incubated for 12 days, at which time the samples were harvested and assayed for infectious virus by plaque assay [63].

### Statistical analysis

Logarithm base 2 (log_2_ transformed) values were used for evaluation of observed viral load data to reduce the effect of very low or very high values which might bias the mean. Statistical analysis indicated log normal distribution of the viral load data; the two-sample T-test was used to determine the difference in geometric means for adjusted viral loads between groups according to viral strain. The Wilcoxon rank-sum test was used to determine the difference in persistent infections at day 270 by different viral strains. The Z test for the comparison of proportion was used to evaluate differences in the frequency of tumor formation between groups exposed to different viral strains and the proportional hazards regression test to assess differences in survival times of animals that developed tumors between groups. Statistical analyses were performed using statistical software SAS version 9.3. A *p*-value of ≤0.05 was considered statistically significant.

## Supporting Information

Figure S1 **SV40 microRNA mutants for strains 776 and SVCPC.** Coding sequences of the portion of T-antigen that represents SVpre-miRNA (shaded) plus the adjacent region are shown for wild-type virus 776-WT T-antigen (GenBank accession No. AAB59924.1) and SVCPC-WT T-antigen (GenBank accession No. AAD39001.1) and for mutants 776-SM1 and SVCPC-SM2. The SV40 miRNA mutant derived from SV40-776 virus (776-SM1) was obtained from Christopher Sullivan. The wobble point mutations introduced to destroy the secondary structure are in boldface. The dot (.) means identity, the dash (–) deleted nucleotide. One-letter amino acid abbreviations (in italics) of 776-WT and SVCPC-WT T-antigens are included on the two bottom lines. The dot (.) means identity, the dash (–) deleted amino acid.
(DOCX)

Table S1 **Dynamics of establishment of SV40 viral persistence in the kidney.** The ratios of observed viral loads in the kidney compared to those in the liver or spleen increased gradually over time with each virus. The kidney∶liver ratios from day 3 to day 45 increased 3.8- to 10-fold, with the largest increase displayed by the microRNA mutant 776-SM1. The kidney∶spleen ratios from day 3 to day 45 also increased, as virus levels in the spleen became very low or undetectable. The numbers of animals tested for each virus per time point is shown in Table 1 (usually n = 4).
(DOCX)
