# Supplementary material for: Viral MicroRNA Effects on Pathogenesis of Polyomavirus SV40 Infections in Syrian Golden Hamsters
Source: PLoS Pathog. 2014 Feb 6;10(2):e1003912. doi: 10.1371/journal.ppat.1003912 (PMC3916418; doi:10.1371/journal.ppat.1003912)
Supplement: Figure S1 — SV40 microRNA mutants for strains 776 and SVCPC. Coding sequences of the portion of T-antigen that represents SVpre-miRNA (shaded) plus the adjacent region are shown for wild-type virus 776-WT T-antigen (GenBank accession No. AAB59924.1) and SVCPC-WT T-antigen (GenBank accession No. AAD39001.1) and for mutants 776-SM1 and SVCPC-SM2. The SV40 miRNA mutant derived from SV40-776 virus (776-SM1) was obtained from Christopher Sullivan. The wobble point mutations introduced to destroy the secondary structure are in boldface. The dot (.) means identity, the dash (–) deleted nucleotide. One-letter amino acid abbreviations (in italics) of 776-WT and SVCPC-WT T-antigens are included on the two bottom lines. The dot (.) means identity, the dash (–) deleted amino acid. (DOCX) [file ppat.1003912.s001.docx]

**Figure S1**

2863

↓

776-WT AGAACATGGAAGACTCAGGGCATGAAACAGGCATTGATTCACAGTCCCAAGGCTCATTTC

776-SM1 ....**T**.....**G**..**T**..**T**..**T**.....**G**..**C**..**T**........**T**.....**G**..**G**..**T**..**G**....

SVCPC-WT **..............................................**T**............**.

SVCPC-SM2 ....**T**.....**G**..**T**..**T**..**T**.....**G**..**C**..**T**........**T**.....**G**..**G**..**T**..**G**....

776-WT *N M E D S  G H E T G I D S  Q S  Q G S  F Q*

SVCPC-WT *. . . . . . . . . . . . . . . . . . . .*

2752

↓

776-WT AGGCCCCTCAGTCCTCACAGTCTGTTCA---------TGATCATAATCAGCCATACCACA

776-SM1 .**A**..**T**..**G**.....**T**..**G**...........---------.......................

SVCPC-WT ...........C..........CTCA..GTCTGTTCA..................T....

SVCPC-SM2 .**A**..**T**..**G**...C.**T**..**G**.....CTCA..GTCTGTTCA..................T....

776-WT *A P Q S S  Q S  V H - - - D H N Q P Y H I*

SVCPC-WT *. . . P . . . S Q S V H . . . . . . . .*
