# Supplementary material for: Viral MicroRNA Effects on Pathogenesis of Polyomavirus SV40 Infections in Syrian Golden Hamsters
Source: PLoS Pathog. 2014 Feb 6;10(2):e1003912. doi: 10.1371/journal.ppat.1003912 (PMC3916418; doi:10.1371/journal.ppat.1003912)
Supplement: Table S1 — Dynamics of establishment of SV40 viral persistence in the kidney. The ratios of observed viral loads in the kidney compared to those in the liver or spleen increased gradually over time with each virus. The kidney∶liver ratios from day 3 to day 45 increased 3.8- to 10-fold, with the largest increase displayed by the microRNA mutant 776-SM1. The kidney∶spleen ratios from day 3 to day 45 also increased, as virus levels in the spleen became very low or undetectable. The numbers of animals tested for each virus per time point is shown in Table 1 (usually n = 4). (DOCX) [file ppat.1003912.s002.docx]

| Table S1. Dynamics of establishment of SV40 viral persistence in the kidney. | | | |
| --- | --- | --- | --- |
|  |  | Ratios of viral loads (SV40 DNA copies/10^4^ cells) | |
| Virus | Days p.i. | Kidney:Liver | Kidney:Spleen |
| 776-WT | 3 | 0.94 | 5.65 |
|  | 7 | 1.18 | 5.15 |
|  | 14 | 2.00 | 85.3 |
|  | 28 | 1.16 | – |
|  | 45 | 3.77 | – |
|  |  |  |  |
| 776-SM1 | 3 | 0.07 | 3.9 |
|  | 7 | 0.17 | 18.1 |
|  | 14 | 0.32 | 27.8 |
|  | 28 | 0.79 | 72.0 |
|  | 45 | 0.70 | 50.5 |
|  |  |  |  |
| SVCPC-WT | 3 | 0.08 | 1.14 |
|  | 7 | 0.18 | 1.03 |
|  | 14 | 0.08 | 5.85 |
|  | 28 | 0.11 | 2.08 |
|  | 45 | 0.30 | 8.8 |
|  |  |  |  |
| SVCPC-SM2 | 3 | 0.12 | 1.03 |
|  | 7 | 0.22 | 6.83 |
|  | 14 | 0.14 | 12.2 |
|  | 28 | 0.35 | 8.65 |
|  | 45 | 0.54 | 61.4 |
